# Proteins associated with the doubling time of the NCI-60 cancer cell lines

**DOI:** 10.1186/s13008-017-0032-y

**Published:** 2017-08-29

**Authors:** Michael Polymenis

**Affiliations:** Department of Biochemistry and Biophysics, Texas A&M University, 2128 TAMU, College Station, TX 77843 USA

**Keywords:** EMT, E2F, MYC

## Abstract

The varied nature of human cancers is recapitulated, at least to some extent, in the diverse NCI-60 panel of human cancer cell lines. Here, I used a basic, continuous variable of proliferating cells, their doubling time, to stratify the proteome across the NCI-60 cell lines. Among >7000 proteins quantified in the NCI-60 panel previously, the levels of 84 proteins increase in cells that proliferate slowly. This set overlapped with the hallmark molecular signature “epithelial-mesenchymal transition (EMT)” (p value = 1.1E−07). Conversely, the levels of 105 proteins increased in cells that proliferate faster and overlapped with the molecular signatures for “MYC targets V1” (p value = 3.8E−38) and “E2F targets” (p value = 2.4E−34). These data for the first time identify proteins whose levels are dynamically associated with doubling time, but not necessarily with cancer type origins, and argue for the incorporation of doubling time measurements in cell line-based profiling studies.

**Electronic supplementary material:**

The online version of this article (doi:10.1186/s13008-017-0032-y) contains supplementary material, which is available to authorized users.

## Text

Much of the molecular characterization of cancer cells tends to be categorical in nature, probably because there are many different types of cancer. Consequently, when biomolecules are measured in cancer cells, they are often reported in categorical terms. For example, countless studies report on proteins whose levels or activity may be high in one cancer but very low or absent in other types of cancer or normal cells. Such approaches are valuable, pointing to molecular signatures unique to specific cancers. However, they also miss molecular variables that are continuous in nature, not discreet. The molecular characterization of the NCI-60 cell lines is extensive. Proteomic variation is of special interest for two reasons: First, proteins represent the vast majority of targets of anticancer drugs. Second, differential protein expression underpins the behavior of the different cancers represented in the panel [1–4]. The overwhelming emphasis and underlying assumptions in such comparative profiling approaches are that strong cell line clustering of protein levels provides biomarkers that are specific to particular tumors. But the different cancer cell lines in the NCI-60 panel also display distinct cell proliferation rates, or doubling times (Td), ranging from 17 to 80 h [5, 6]. However, the different doubling times of the cell lines are rarely taken into consideration in comparative profiling analyses. The NCI-60 panel is the only system for which systematic measurements of the proteome and cell cycle times are available for such a wide range of human cells, cancerous or not. The extent to which differences in doubling times among the NCI-60 cancer cell lines are related to the observed differences in protein levels has not been explored.

**Fig. 1:**
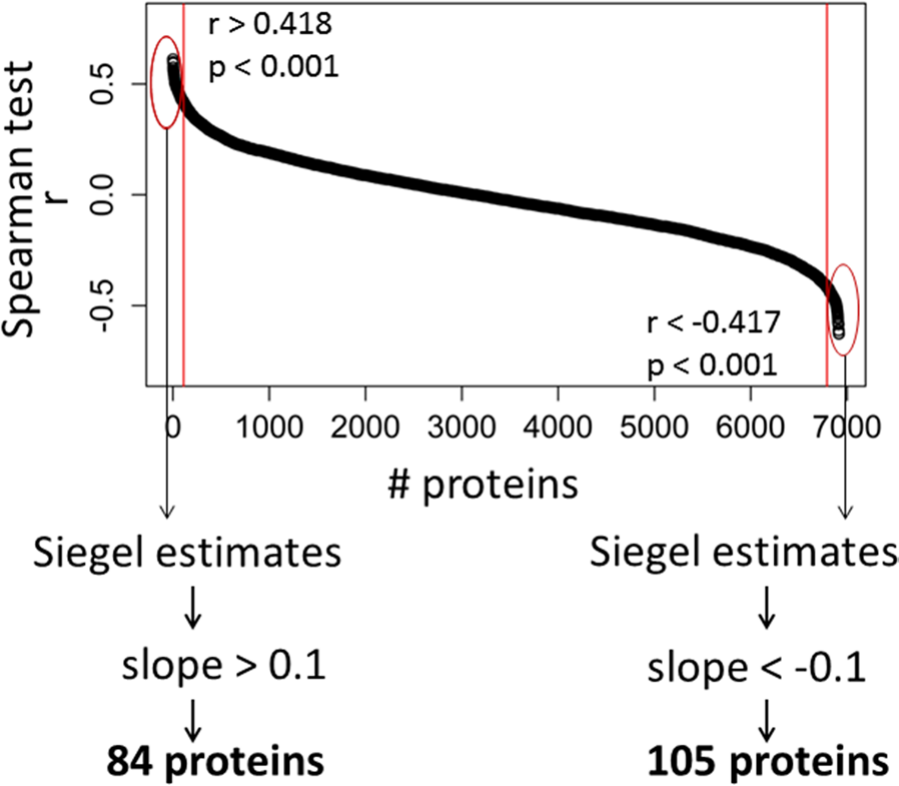
Identifying proteins associated with the growth rate of cancer cells. The Spearman rank correlation coefficient r (*y-axis*) for the association between the levels of each protein and the doubling time (Td) in the NCI-60 panel. The *red vertical lines* indicate the p value cutoff (p < 0.001). From these Td-correlated proteins, we estimated the slope of the linear regression model and retained cases with a |slope| >0.1. The 84 proteins associated with long Td are shown in Additional file 1: Table S1, while the 105 proteins associated with short Td are shown in Additional file 1: Table S2. The gene ontology (GO) categories of biological processes associated with these proteins are shown in Additional file 1: Tables S3 and S4

Quantitative immunoblotting against a limited number of target proteins was originally used to query the proteome of the NCI-60 panel [2, 3], followed by comprehensive mass spectrometry approaches [1]. Accurate values for the doubling time of each cell line are available from NCI’s Developmental Therapeutics Program (DTP), representing the average of multiple independent estimates [5, 6]. To test for positive or negative association between the levels of each protein and the doubling time, I used Spearman’s non-parametric, distribution-free test for independence based on ranks. A correlation matrix of all the protein levels in each cell line against the doubling time of that cell line was generated, using the *rcor* function of the R language package *Hmisc*. This analysis yielded the Spearman rank correlation coefficient r and the corresponding p value for the association between the levels of each protein and the doubling time (Td), shown in Fig. 1. The levels of the vast majority of proteins, including proteins with housekeeping functions, did not correlate strongly with the doubling time (Td) of the cell populations. However, the levels of two groups of proteins were highly correlated with Td, either positively or negatively with (|r| > 0.41, p < 0.001; see Fig. 1). The above analysis alone does not offer a view of the magnitude of the correlation: protein levels may only change within a very narrow range. For those proteins whose levels were correlated to Td, the slope of the regression of protein levels on Td would yield the magnitude of the effect. Hence, linear regression models for each protein whose levels were highly correlated with Td were constructed (Fig. 1). For the regression analyses, the non-parametric, Siegel repeated median estimates [7] were used to obtain the slopes and intercepts of the linear models (with the R language package *mblm*).

Overall, from >7000 proteins that entered this analysis, from the significance cutoffs we applied (p < 0.001), one would have expected <8 proteins by chance to be correlated with long, or short, Td. However, we found that the levels of 84 were not only correlated with Td but also the slope of the regression of those protein levels on Td was greater than 0.1 (Fig. 1; Additional file 1: Table S1). The levels of those proteins increase in cells that have a higher Td and proliferate slowly. To gauge the physiological significance of these findings, the interactive and freely available *Enrichr* platform [8, 9] was used for enrichment analysis for biological processes and molecular pathways. The set of 84 proteins whose levels increase in cells that proliferate slower were enriched for the related biological processes “collagen fibril organization” and “extracellular matrix organization” (GO: 0030199 and GO: 0030198; see Additional file 1: Table S3). The same group of proteins also overlapped significantly with the hallmark (Molecular Signatures Database; MSigDB [10]) sets for “epithelial-mesenchymal transition (EMT)” (p value = 1.1E−07) and “protein secretion” (p value = 1.1E−06). The slowest proliferating cell lines may resemble differentiated cells more than other cell lines. However, it did not seem to be the case that a specific cancer type is enriched in the slower growing cell lines. For example, the six slowest proliferating cell lines were from non-small cell lung (LC: HOP-92), Renal (RE: A498 and RXF393), central nervous system (CNS: SNB-75) and breast (BR: BT-549) cancers. Some of the fastest proliferating cell lines were also from the same types of cancer (LC: NCI-H460 and A549, RE: 786-0, CNS: U251). Activation of the EMT program is known to play a key role in cancer cell invasion and metastasis [11, 12]. These data suggest that that induction of the EMT program is associated with lower rates of cell proliferation, which may be a property of metastasizing cells. While it is not known whether the slower growing NCI60 cell lines are more prone to undergo EMT in orthotopic models, it is known that overexpression of certain proteins is sufficient to cause EMT, slow tumor growth, and late steps in metastasis [13].

Conversely, the levels of 105 proteins regressed on Td with a slope lower than −0.1. The levels of those proteins increase in cancer cells that proliferate fast and have a lower Td (Fig. 1; Additional file 1: Table S2). The set of 105 proteins whose levels increase in cells that proliferate faster were enriched for the biological processes “positive regulation of telomerase RNA localization to Cajal body” and “DNA strand elongation involved in DNA replication” (GO: 19048746 and GO: 0006271; see Additional file 1: Table S4). The same set of 105 proteins also overlapped to a very significant extent with the hallmark sets for “MYC targets V1” (p value = 3.8E−38) and “E2F targets” (p value = 2.4E−34). Therefore, many proteins in this set control cell growth and the timing of initiation of cell division at the G1/S transition. In this set of 105 proteins, five are already classified as oncogenic in MSigDB (DDX5, DDX6, ATIC, HSP90AB1, CBFB). The overwhelming majority of the proteins in this set are involved in cell division and cell growth. Cell growth (increase in mass) determines the rate at which cells proliferate [14], but how cells couple their growth with their division is not well understood. These data underscore the fundamental and cooperative role of the MYC and E2F networks in the physiological coupling between growth and division, to control rates of cell proliferation [15].

In conclusion, the above analysis argues that it is reasonable to incorporate the doubling time as a key phenotypic variable when profiling the behavior of different cancer cell lines. Specifically, protein biomarkers that emerge from the profiling of these cell lines may be a reflection of the doubling time of the particular cell line, and not necessarily attributed to the origin of the cancer type in question. Lastly, a surprising result of this study is that the levels of ‘housekeeping’ gene products (e.g., ribosomal proteins) are not dynamically associated with growth rate. Instead, it was the levels of other functional groups of proteins (e.g., the MYC and E2F networks) that appear to be more closely associated with rates of cell proliferation.

## Additional file

**Additional file 1.** Additional tables.
